# Assessing the Effects of Moderate to High Dosage of Astaxanthin Supplementation on Lipid Profile Parameters—A Systematic Review and Meta-Analysis of Randomized Controlled Studies

**DOI:** 10.3390/ph18081097

**Published:** 2025-07-24

**Authors:** Lucas Fornari Laurindo, Victória Dogani Rodrigues, Dennis Penna Carneiro, Luiz Sérgio Marangão Filho, Eliana de Souza Bastos Mazuqueli Pereira, Ricardo José Tofano, Eduardo Federighi Baisi Chagas, Jesselina Francisco dos Santos Haber, Flávia Cristina Castilho Caracio, Letícia Zanoni Moreira, Vitor Engrácia Valenti, Sandra Maria Barbalho

**Affiliations:** 1 Laboratory for Systematic Investigations of Diseases, Department of Biochemistry and Pharmacology, School of Medicine, Universidade de Marília (UNIMAR), Marília 17525-902, SP, Brazil; 2 Postgraduate Program in Structural and Functional Interactions in Rehabilitation, School of Medicine, Universidade de Marília (UNIMAR), Marília 17525-902, SP, Brazil; 3 Department of Biochemistry and Pharmacology, School of Medicine, Faculdade de Medicina de Marília (FAMEMA), Marília 17519-030, SP, Brazil; 4 Department of Biochemistry and Pharmacology, School of Medicine, Universidade de Marília (UNIMAR), Marília 17525-902, SP, Brazil; 5 Faculty of Philosophy and Sciences, Universidade Estadual Paulista (UNESP), Marília Campus, Marília 17525-900, SP, Brazil; 6 Department of Biochemistry and Nutrition, School of Food and Technology of Marília (FATEC), Marília 17500-000, SP, Brazil

**Keywords:** astaxanthin, carotenoid, lipids, low-density lipoprotein cholesterol, high-density lipoprotein cholesterol, triglycerides, total cholesterol, cholesterol

## Abstract

**Background/Objectives:** Astaxanthin, a xanthophyll carotenoid, has garnered significant interest due to its benefits with regard to dyslipidemia. This multifaceted functional food ingredient modulates several key enzymes associated with lipid regulation, including HMG-CoA reductase, CPT1, ACCβ, and acyl-CoA oxidase. It influences key antioxidant molecular pathways like the Nrf2, limiting dyslipidemia occurrence and regulating liver cholesterol uptake through the modulation of liver lipid receptors. Due to the current lack of systematic reviews and meta-analyses assessing moderate to high dosages (6–24 mg/d) of astaxanthin supplementation on lipid dysregulation, the present manuscript aims to fill this gap in the literature. **Methods:** Following the PRISMA guidelines, we included eight studies comprising eleven results from the PubMed, Springer Link, Science Direct, Cochrane, and Google Scholar databases. The Jamovi (Version 2.6.26, Solid) software was utilized for statistics. Our primary objective was to assess in detail the effects of astaxanthin on LDL-C, HDL-C, triglyceride, and total cholesterol levels. **Results:** The meta-analysis concludes positive effects of astaxanthin (6–20 mg/d) on HDL-C (0.4200; 95% CI: 0.1081 to 0.7319) and triglyceride (−0.3058; 95% CI: −0.5138 to −0.0978) levels. Unfortunately, astaxanthin (6–20 mg/d) does not appear to significantly influence LDL-C (−0.0725; 95% CI: −0.3070 to 0.1620) and total cholesterol (−0.0448; 95% CI: −0.3369 to 0.2473) levels. Regarding HDL-C, improvements were observed from 55 ± 8 mg/dL (pre-intervention) to 63 ± 8 mg/dL (post-intervention) (*p* < 0.01) in the 12 mg/d of astaxanthin groups. In the assessment of triglyceride levels, results show a decrease from 151 ± 26 mg/dL (pre-intervention) to 112 ± 40 mg/dL (post-intervention) (*p* < 0.01) for 18 mg/d astaxanthin supplementation. **Conclusions:** Further research is necessary to fully harness the potential of astaxanthin, which includes assessing astaxanthin in different subsets of patients, using a GWAS, and in combination with other nutraceuticals to understand the compound’s effectiveness with regard to varying health conditions, genetic and epigenetic factors, and synergistic effects with other compounds.

## 1. Introduction

Dyslipidemia refers to abnormal levels of blood lipids. It is often characterized by decreased levels of high-density lipoprotein cholesterol (HDL-C) and elevated low-density lipoprotein cholesterol levels (LDL-C), accompanied by increased triglyceride and total cholesterol levels. This condition increases the risk for cardiovascular diseases and outcomes, including coronary artery disease and atherosclerosis, ultimately leading to increased morbidity and mortality rates [1]. Currently, many pharmacological interventions are being assessed for lipid-lowering effects, including 3-hydroxy-3-methylglutaryl-coenzyme A reductase (HMG-CoA reductase) inhibitors like statins [2]; selective cholesterol absorption inhibitors like ezetimibe [3]; proprotein convertase subtilisin/kexin type 9 inhibitors (PCSK9is) like alirocumab and evolocumab [4]; bile acid sequestrants like cholestyramine, colestipol, and colesevelam [5]; and nutritional supplements [6]. In this context, medicinal plants, functional foods, and bioactive compounds are prominent because they present high efficacy with lower prices and fewer adverse effects, leading to an overall metabolic health amelioration and inducing not only lipid-level control, but also reducing blood pressure and improving glucose homeostasis, ultimately leading to a decreased cardiovascular risk [7].

**Figure 1:**
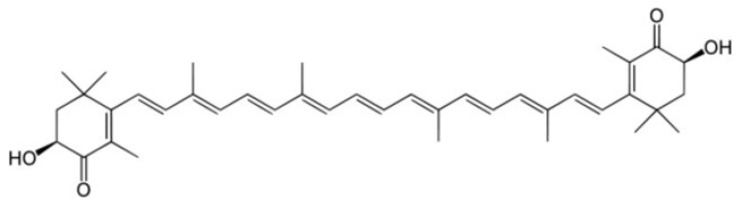
Molecular structure of astaxanthin.

Astaxanthin, a reddish-orange-hued xanthophyll carotenoid, is derived from many sources, including plants, yeast, lichens, fungi, and microalgae. This compound has high antioxidant, anti-inflammatory, antidiabetic, and anti-aging properties and has been proven to enhance cardiovascular health [8,9,10]. Research has also indicated that astaxanthin may present potent lipid-lowering effects. It can improve lipid parameters by decreasing lipid peroxidation, enhancing antioxidant defense, and improving the genetic expression of various enzymes related to lipid metabolism, including the redox-sensitive enzymes. Additionally, astaxanthin elevates messenger ribonucleic acid (mRNA) levels of the low-density lipoprotein (LDL) receptor (LDLR) in the liver, stimulating enhanced cholesterol uptake by hepatocytes, and modulates the levels of HMG-CoA reductase enzyme, which is the limiting-phase enzyme for cholesterol synthesis. Astaxanthin also modulates sterol-regulatory element-binding protein 2 (SREBP2) levels, thereby balancing lipid levels. In addition, astaxanthin modulates the levels of the enzymes carnitine palmitoyl transferase 1 (CPT1), acetyl-CoA carboxylase β (ACCβ), and acyl-CoA oxidase (ACOX), which are critical to fatty-acid β oxidation [11,12]. Finally, astaxanthin has also been reported to enhance the expression of nuclear factor erythroid 2-related factor 2 (Nrf2), which in turn reduces plasma triglycerides and increases ACOX mRNA levels [13]. Figure 1 depicts the molecular structure of astaxanthin.

**Figure 2:**
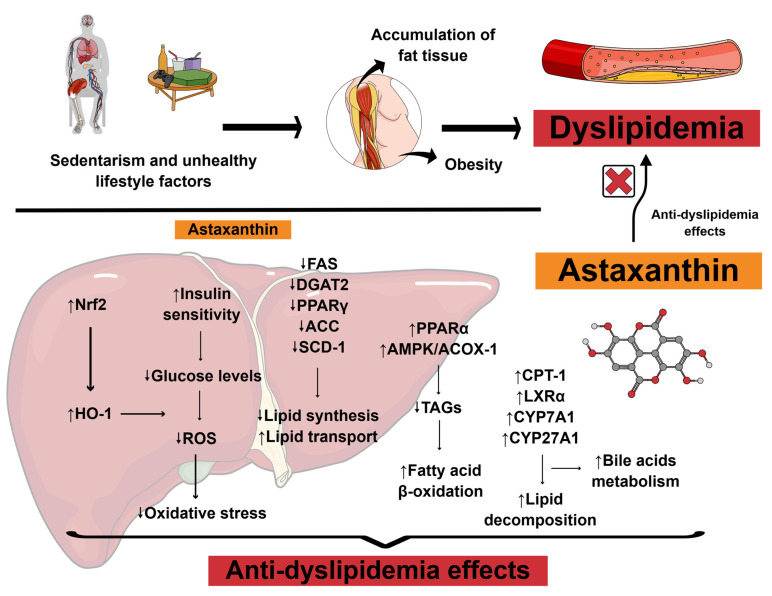
Scientific illustration depicting the anti-dyslipidemia effects associated with astaxanthin supplementation and their potential mechanisms of action. Abbreviations: ↑, increase; ↓, decrease; ACC, acetyl-CoA carboxylase; ACOX-1, acyl-CoA oxidase 1; AMPK, adenosine monophosphate-activated protein kinase; CPT-1, carnitine palmitoyltransferase I; CYP27A1, sterol 27-hydroxylase; CYP7A1, cholesterol 7α-hydroxylase; DGAT2, diacylglycerol O-acyltransferase 2; FAS, fatty acid synthase; HO-1, heme oxygenase-1; LXRα, liver X receptor alpha; Nrf2, nuclear factor erythroid 2-related factor 2; PPARα, peroxisome proliferator-activated receptor alpha; PPARγ, peroxisome proliferator-activated receptor gamma; ROS, reactive oxygen species; SCD-1, stearoyl CoA desaturase 1; TAGs, triacylglycerols. Based on Gao et al. (2025) [16]. Created using https://mindthegraph.com/ (accessed on 14 July 2025).

Nutritional management of dyslipidemia has been shown to improve overall metabolic health in patients, even without the use of lipid-lowering synthetic medications. These dietary strategies modulate relevant biomarkers involved in lipid production, and when combined with exercise regimens, medical nutrition therapies might be even more potentiated [14]. Bioactive compounds have gained significant interest in this context due to their multifaceted health effects [15]. Figure 2 illustrates the potential anti-dyslipidemia effects associated with astaxanthin supplementation and their corresponding mechanisms of action.

Several previous studies have conducted systematic evaluations of astaxanthin as a functional supplement that affects lipid status. Wan et al. [17] reviewed the beneficial effects of some antioxidants, including astaxanthin, on patients’ overall cardiovascular risk. However, their study did not assess astaxanthin alone, and their analysis lacked a sufficient in-depth exploration of astaxanthin supplementation against dysregulated lipids, as they utilized data on omega-3 fatty acids, lycopene, and other omegas regarding various cardiovascular disease risk factors, including blood pressure and glucose levels. Additionally, they did not restrict data on astaxanthin dosage; therefore, they did not strictly assess moderate to high dosages of astaxanthin, which limits the generalizability of the results to low dosages that may not fully represent the efficacy of the nutraceutical. The same scenario occurred with Leung et al. [18], who reviewed the beneficial effects of astaxanthin on metabolic syndrome risk factors without any restrictions on dosage comparisons and outcome measurements. The present systematic review and meta-analysis aim to fill this gap in the literature by reporting promising results from moderate- to high-dose astaxanthin supplementation in improving lipid parameters in human subjects.

## 2. Materials and Methods

### 2.1. Focused Question

This systematic review and meta-analysis were designed to answer the question, “*What are the effects of astaxanthin on improving the lipid profile of human subjects?*”

### 2.2. Language

This review included only trials published initially in English. Although this might be considered a bias, a previous review paper noted no evidence of a systematic bias in literature reviews due to English language restrictions [19].

### 2.3. Literature Search and Databases

We investigated studies searching in the PubMed, Springer Link, Science Direct, Cochrane, and Google Scholar databases. We utilized the mesh terms “astaxanthin,” “lipid profile,” “triglycerides,” “low-density lipoprotein cholesterol,” “high-density lipoprotein cholesterol,” “lipids,” and “total cholesterol.” Boolean operators “AND” or “OR” were utilized to refine the literature search process. All articles acknowledged were exported to the Rayyan QCRI program (Qatar Computing Research Institute, Doha, Qatar) to eliminate duplicates. The software screened the studies by reading the title and abstract. L.F.L. and V.E.V. completed the suitability stage by reading the manuscript selected after duplication screening. Another reviewer (S.M.B.) decided whether a specific study should be included in the event of disagreement. The authors evaluated the possibility of conducting a meta-analysis of the group after assessing all the included studies (that met the eligibility criteria). There is no registration number for this systematic review and meta-analysis.

### 2.4. Study Selection

All the included studies originated from reputable peer-reviewed journals. These were published from the beginning of the database until May 2025. The PICOS (Population, Intervention, Comparison, Outcomes, and Study Design) elements below guided the inclusion and exclusion criteria:

(P) Patients were older than 18 years old.

(I) The intervention group ought to receive moderate to high doses of astaxanthin (6–24 mg/d) as an intervention.

(C) We included studies that evaluated subjects who received a placebo for the effect of comparisons between groups.

(O) LDL-C, HDL-C, triglyceride, and total cholesterol levels were outcomes of interest for this systematic review and meta-analysis.

(S) All included studies were randomized and placebo-controlled or had a control group. We only included articles retrieved from reputable peer-reviewed journals and written in English. We excluded conference papers, abstracts, master’s dissertations, doctoral theses, descriptive studies, commentaries, case studies, editorials, and reviews. However, previous reviews on astaxanthin’s general effects were utilized to build some sections of our present manuscript, including the Introduction. The time range for the studies included the years until May 2025.

### 2.5. Data Extraction

We gathered data concerning the author, year of publication, study design, number of study participants, demographic information, and data on the intervention protocols of the respective studies included. This information was extracted from primary studies and presented in a table. All important missing data were requested by contacting the corresponding study authors. Two reviewers (E.F.B.C. and V.D.R.) finished this stage independently. We utilized Web Plot Digitizer^®^ (https://automeris.io/) to extract the data presented in published graphs from the primary articles when the corresponding authors did not respond. Data on lipid profiles were charted as the mean and standard deviation. Values presenting with “standard error” or “confidence intervals” in the primary studies were converted to standard deviation.

### 2.6. Search and Selection of Relevant Articles

Data was collected following the PRISMA guidelines according to the standards reported by Page et al. The writing of this manuscript’s text also responded to the PRISMA checklist for main text and abstracts [20].

### 2.7. Data Items

We collected mean and standard deviations from the LDL-C, HDL-C, triglyceride, and total cholesterol values. In addition, data concerning participant and intervention profiles, funding sources, and supports across the included articles were obtained from the selected references. At this point, missing or unclear information was discarded.

### 2.8. Quality Assessment

We utilized the *Cochrane Handbook for Intervention Assessments* [21] to evaluate the bias of the included studies. We gathered information regarding question focus, appropriate randomization, allocation blinding, losses of participants, prognostic and demographic characteristics, outcomes, intention-to-treat analysis, sample calculations, and adequate time for follow-up.

### 2.9. Qualitative Analysis

We conducted a narrative synthesis to provide a detailed description of each study’s completion. Each study’s details were introduced in texts and tables. The results for each survey regarding lipid intervention with astaxanthin supplementation were established by comparing the results from the intervention and comparison (placebo) groups.

### 2.10. Synthesis of Results and Summary Measures

The statistical analysis used Jamovi-open statistics software (Version 2.6.26, Solid). We utilized a *p*-value of less than 0.05 as the threshold for statistical significance. To conduct this meta-analysis, we used the standardized mean difference as the outcome measure, and a random-effects model was applied to the data [22]. Heterogeneity (i.e., tau^2^) was estimated using the DerSimonian–Laird estimator. Additionally, we also reported the Q-test for heterogeneity and the I^2^ statistic. Whether heterogeneity was detected (i.e., tau^2^ > 0, regardless of the results of the Q-test), we predicted the interval for the actual outcomes. We utilized the studentized residuals and Cook’s distances to examine whether the included studies may be outliers and/or influential in the model context. When a study possessed a studentized residual larger than the 100 × (1 − 0.05/(2 × k))^th^ (where k is the number of included results) percentile, this was considered a potential outlier due to deviation from a standard normal distribution (i.e., using the Bonferroni correction test with a two-sided α = 0.05 for n studies that were included in this meta-analysis). In addition, studies were considered influential when they presented a Cook’s distance larger than the median plus six times the interquartile range of the Cook’s distances. To check for funnel plot asymmetry, the rank correlation test and the regression test, using the standard error of the observed outcomes as predictor, were used [23,24].

### 2.11. Certainty Assessment (Levels of Evidence)

We utilized the Grades of Recommendation, Assessment, Development, and Evaluation (GRADE) Working Group [25] to gauge the certainty of the evidence. This analysis includes the study design of randomized trials (strong evidence). We also considered study quality (detailed study methods and execution) and restrictions in the strength of evidence analysis [26]. We executed the GRADEpro GDT v4^®^ (McMaster University, Hamilton, ON, Canada) to elaborate on the summary of the findings table.

## 3. Results and Discussion

**Figure 3:**
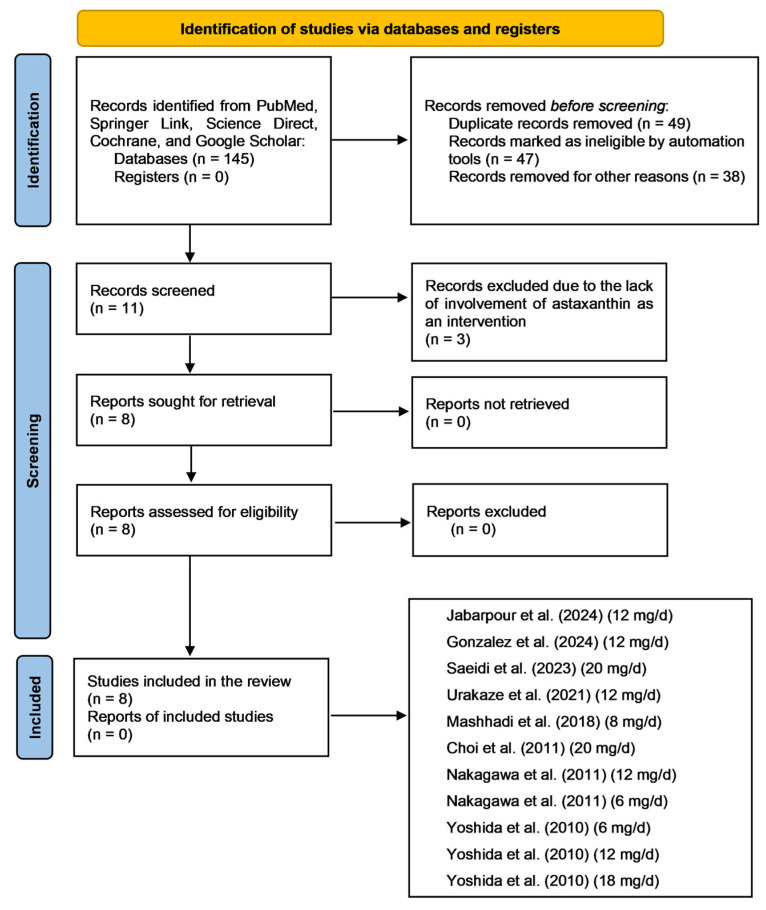
Flow diagram depicting the literature search process following the Preferred Reporting Items for Systematic Reviews and Meta-Analyses (PRISMA) guidelines [20]. Abbreviations: mg, milligram; d, day. The included studies are [27,28,29,30,31,32,33,34].

During the initial identification of the included studies, 145 records were identified from the databases searched. At this stage, 49 records were removed from the sample due to duplication, 47 were identified as ineligible by automation tools, and 38 were excluded for other reasons, including inconsistencies in methodology that conflicted with our selection criteria. Following this stage, 11 records were screened, and 3 additional records were excluded because they lacked involvement of astaxanthin as an intervention. After this, eight records were retrieved. Fortunately, all reports were successfully retrieved and included in the final analysis. Figure 3 illustrates a flow diagram outlining the literature search process and the included studies, adhering to the Preferred Reporting Items for Systematic Reviews and Meta-Analyses (PRISMA) guidelines. Because we included results from two studies in our meta-analysis that utilized different dosages of astaxanthin as interventions, this figure also depicts the different doses used in these two studies [27,28]. Table 1 describes the included studies following the PRISMA guidelines. Table 2 presents the bias assessment for each included study based on the *Cochrane Handbook*.

In a study conducted with polycystic ovary syndrome (PCOS) patients, Jabarpour et al. evaluated the effects of 12 mg/d of astaxanthin on lipid profiles in a triple-blind, randomized, placebo-controlled trial. Twenty-seven patients received astaxanthin (30.36 ± 5.16 years, 26.12 ± 1.56 kg/m^2^) and another twenty-six a placebo (30.84 ± 4.84 years, 26.24 ± 1.59 kg/m^2^) for eight weeks. The results demonstrated significant modulation of the lipid profile within the intervention group, especially in HDL-C (37.61 ± 10.08 mg/dL → 41.80 ± 9.965 mg/dL) and LDL-C (91.27 ± 21.08 mg/dL → 82.52 ± 23.58 mg/dL), with significance levels of *p* = 0.003 and *p* = 0.013, respectively [29]. Although this study has its merits, the results must be interpreted cautiously. The authors did not evaluate the serum or plasma levels of astaxanthin during the intervention, so the patient’s compliance with the intervention was not assessed. However, no adverse effects have been observed. Additionally, the intervention period may be considered short, and the 8-week intervention might have contributed to the insignificant results regarding the effects of astaxanthin on the triglyceride and total cholesterol levels.

In the United States, Gonzalez et al. [30] conducted a randomized, double-blind, placebo-controlled study to evaluate the effects of 12 mg/d of astaxanthin on the health profile of fifteen male patients aged 34.5 ± 7.5 years with a mean body mass index (BMI) of 30.1 ± 2.9 kg/m^2^. Participants were also evaluated through a standardized training program, and the intervention with astaxanthin lasted four weeks. The results did not demonstrate positive outcomes regarding total cholesterol (197.8 ± 29.9 mg/dL → 217.9 ± 33.2 mg/dL, *p* = 0.783), triglycerides (117.7 ± 40.9 mg/dL → 132.5 ± 45 mg/dL, *p* = 0.827), HDL-C (47.1 ± 8.4 mg/dL → 51.9 ± 9.3 mg/dL, *p* = 0.724), or LDL-C (127.7 ± 28.3 mg/dL → 140.5 ± 30.3 mg/dL, *p* = 0.628). Many side effects were observed, including dizziness, headache, tachycardia, palpitations, dyspnea, nervousness, and blurred vision, and the study also has a few limitations that must be thoughtfully addressed. Firstly, these authors did not assess astaxanthin concentrations in blood samples of the tested participants, which limits the understanding of patient compliance during the intervention. Additionally, the short intervention duration of 4 weeks may be insufficient for favorable outcomes. Finally, the male participants were firefighters; therefore, they were considered physically trained before the intervention started, limiting the findings’ generalizability.

In a randomized, placebo-controlled trial involving obese male patients aged 27.6 ± 8.4 years (33.6 ± 1.4 kg/m^2^), Saeidi et al. investigated the effects of high-dose astaxanthin (20 mg/d) for 12 weeks on metabolic parameters. The results demonstrated that the supplemented group achieved better results compared to the placebo group, exhibiting statistically significant decreases in total cholesterol (229.4 ± 5.4 mg/dL → 224.0 ± 5.1 mg/dL), triglycerides (247.8 ± 5.9 mg/dL → 244.1 ± 5.4 mg/dL), and LDL-C (127.5 ± 5.4 mg/dL → 123.4 ± 5.2 mg/dL) and increases in HDL-C (37.8 ± 1.23 mg/dL → 39.8 ± 1.2 mg/dL) levels, with *p* values < 0.05 for all interventions [31]. Although this study included a high dose of astaxanthin and a significant intervention period, it experienced high drop-out rates, which included the withdrawal of eight participants.

In a study involving 23 (46.2 ± 13.7 years, 21 ± 2 kg/m^2^) Japanese patients receiving 12 mg/d of astaxanthin as an intervention for 12 weeks, Urakaze et al. [32] reported that astaxanthin could reduce diabetes and diabetic-related atherosclerosis pathways via glucose- and modified LDL-C-lowering effects. However, the intervention did not provide statistically significant results with regard to plasma lipid dysregulation. Besides the high-dose astaxanthin intervention, patients did not perceive any adverse effects associated with using nutraceuticals. Although this study employed a randomized, double-blind, controlled design, it has some limitations that must be addressed. Firstly, it became unclear whether the differences in diabetic-related outcomes might have influenced lipid levels, since glycemia may be altered despite concomitant changes in lipid outcomes. Therefore, a more extensive intervention might be necessary to address this limitation. Additionally, the study comprised four losses in the intervention group and did not use intention-to-treat analysis, which can limit the generalizability of the results.

In Iran, Mashhadi et al. [33] conducted a double-blind, randomized, placebo-controlled study to assess the effects of astaxanthin supplementation (8 mg/d for eight weeks) on lipid profiles in 22 patients (30 ± 5.11 kg/m^2^) diagnosed with type 2 diabetes mellitus. Besides benefits in diabetic-related parameters, the intervention significantly decreased serum triglyceride levels (156 ± 90 mg/dL → 128 ± 52 mg/dL, *p* = 0.05) without any adverse effects being reported. However, since type 2 diabetes patients may often have hypertriglyceridemia together with uncontrolled glucose metabolism, the authors might have assessed the astaxanthin supplementation in comparison with typical antidiabetic and antilipidemic drugs, including metformin and statins, respectively.

Choi et al. in South Korea evaluated the effects of 20 mg/d of astaxanthin for 12 weeks in fourteen subjects (31.1 ± 9.4 years, 28.1 ± 2.4 kg/m^2^) using a randomized, double-blind, placebo-controlled intervention. The results demonstrated positive outcomes regarding LDL-C serum levels (*p* < 0.05), although the patients experienced fecal color changes and increased bowel movements [34]. Although this study involved an extended intervention period and utilized a very high dose of astaxanthin as an intervention, it did not comprise an elevated sample size. However, the minimal number of participants was calculated prior to the intervention.

In a double-blind, randomized, placebo-controlled trial involving 20 Japanese individuals as the intervention group, Nakagawa et al. [27] evaluated the effects of astaxanthin supplementation at 6 mg/d (56.3 ± 6.6 years, 27.4 ± 2.2 kg/m^2^) and 12 mg/d (56.1 ± 5.1 years, 27.6 ± 2.1 kg/m^2^) once daily for 12 weeks on lipid dysregulation. Although some patients showed changes from the baseline, primarily regarding LDL-C and total cholesterol levels, these were insignificant and did not reach a statistically significant level. Therefore, the intervention appeared to have no effect on lipid control. However, this study possesses some strengths that must be addressed. Firstly, the use of two dosages as a comparison between intervention and placebo patients increases the generalizability of the findings and significantly opens the door for future research endeavors assessing astaxanthin. Additionally, this study had no losses of participants.

Finally, Yoshida et al. [28] evaluated the effects of 6 mg/d (47 ± 7 years, 23.6 ± 3.2 kg/m^2^), 12 mg/d (42.8 ± 8.8 years, 23 ± 2.2 kg/m^2^), and 18 mg/d (43.8 ± 10.4 years, 23.9 ± 7 kg/m^2^) of astaxanthin supplementation on lipids in hypertriglyceridemic Japanese patients using a double-blind, placebo-controlled, randomized clinical design. Astaxanthin demonstrated significant outcomes regarding lipid control, especially while assessing triglyceride (151 ± 23 mg/dL → 125 ± 41 mg/dL, *p* < 0.05) and HDL-C (51 ± 11 mg/dL → 56 ± 11 mg/dL, *p* < 0.01) levels in the 6 mg/d group, triglyceride (147 ± 21 mg/dL → 110 ± 44 mg/dL, *p* < 0.05) and HDL-C (55 ± 8 mg/dL → 63 ± 8 mg/dL, *p* < 0.01) levels in the 12 mg/d group, and triglyceride (151 ± 26 mg/dL → 112 ± 40 mg/dL, *p* < 0.01) and HDL-C (51 ± 6 mg/dL → 55 ± 8 mg/dL, *p* < 0.05) levels in the 18 mg/d group. The intervention did not cause any adverse effects, and all participants finished the trial. This study has some strengths that must be evaluated. Firstly, the sample comprised significantly similar baseline groups for intervention and control scenarios, and the researchers utilized three different doses of astaxanthin to assess the complete scenario of this nutraceutical supplement’s effects on lipids. However, they did not report calculation of the minimum number of participants via sample calculations. Therefore, the results might not be generalizable.

### 3.1. Meta-Analysis of the Effects of Astaxanthin on LDL-C, HDL-C, Total Cholesterol, and Triglyceride Levels

The following subsections will delve into the results from the meta-analysis conducted with the studies included in this review. Despite the non-significant LDL-C and total cholesterol changes in our analysis regarding astaxanthin supplementation in patients’ lipid profiles, there were positive outcomes for HDL-C and triglyceride levels. All subsections contain a forest plot. The funnel plots are presented further in the article. Figures S1 and S2 in the Supplementary Material indicate no substantial differences in the included studies’ participants for age and BMI, respectively. Unfortunately, due to a lack of data regarding age, the forest plot lacks information regarding the surveys by Saeidi et al. [31] and Gonzalez et al. [30] in the final analysis.

### 3.2. Results of the Meta-Analysis for Low-Density Lipoprotein Cholesterol

**Figure 4:**
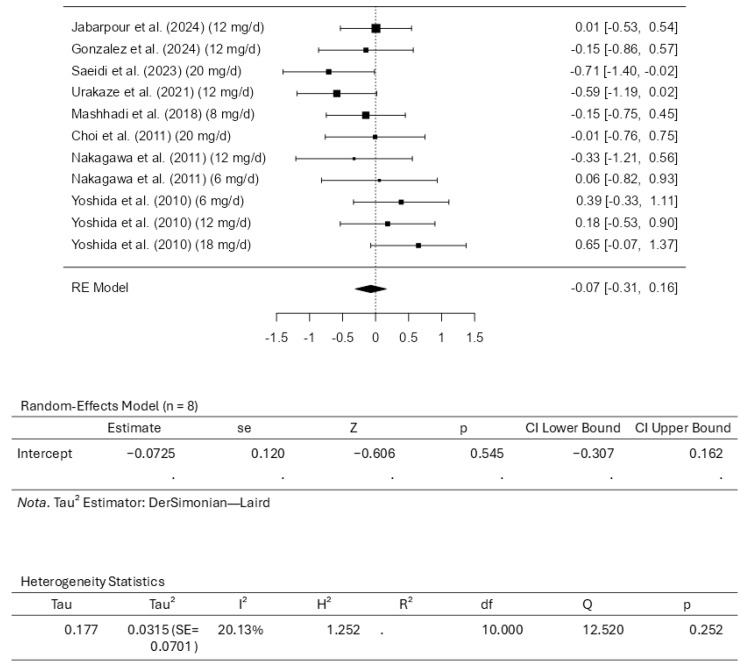
Forest plot depicting the sample setting for low-density lipoprotein cholesterol (LDL-C). The included studies are [27,28,29,30,31,32,33,34].

Eight studies, comprising eleven results, were included in the meta-analysis regarding LDL-C intervention with astaxanthin. The observed standardized mean differences range from −0.7092 to 0.6495, and most estimates were negative (55%). On the other hand, while assessing the estimated average standardized mean difference, it was calculated as −0.0725 (95% CI: −0.3070 to 0.1620) based on the random-effects model. Therefore, the average outcome does not differ significantly from zero (z = −0.6059, *p* = 0.5446). The Q-test showed no significant heterogeneity in the outcomes (Q(10) = 12.5200, *p* = 0.2518, tau^2^ = 0.0315, I^2^ = 20.1278%). A 95% prediction interval for the actual outcomes is given from −0.4919 to 0.3470. Therefore, in some studies, the actual outcome may be positive, although the average outcome is estimated to be negative. Examining the studentized residuals revealed that none of the studies has a value larger than ±2.8376; therefore, there is no indication of outliers in the context of this model. The Cook’s distances indicate that none of the studies could be overly influential. Finally, the funnel plots indicate no plot asymmetry (*p* = 0.5423 and *p* = 0.5965, respectively), as measured by the rank correlation or the regression test. Figure 4 illustrates the forest plot and other results for the meta-analysis assessing LDL-C levels throughout the included studies.

### 3.3. Results of the Meta-Analysis for High-Density Lipoprotein Cholesterol

**Figure 5:**
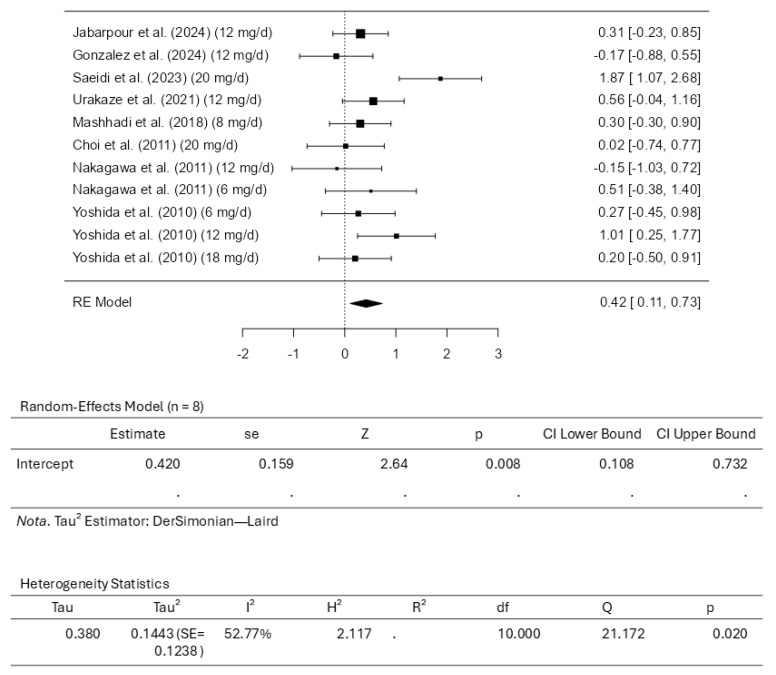
Forest plot depicting the sample setting for high-density lipoprotein cholesterol (HDL-C). The included studies are [27,28,29,30,31,32,33,34].

Eight studies comprising eleven results were included in the meta-analysis for HDL-C intervention using astaxanthin. The results observed a standardized mean difference from −0.1656 to 1.8731. The analysis demonstrated that most estimates were positive (82%). In this scenario, the estimated average standardized mean difference based on the random-effects model was 0.4200 (95% CI: 0.1081 to 0.7319). Therefore, it is worth noting that the average outcome differed significantly from zero (z = 2.6393, *p* = 0.0083). The Q-test revealed that the outcomes appear heterogeneous, with a Q(10) of 21.1719 (*p* = 0.0199, tau^2^ = 0.1443, I^2^ = 52.7675%). Using a 95% prediction interval, the outcomes are given from −0.3872 to 1.2272. This means that although the average outcome is estimated to be positive, the actual outcome may be negative in some studies. An examination of the studentized residuals revealed that the survey conducted by Saeidi et al. [31], which assessed the dosage of 20 mg/d of astaxanthin, has a value larger than ±2.8376 and may represent a potential outlier in the context of this model. Cook’s distances indicated that none of the studies could be overly influential. Further analyses revealed that neither the rank correlation nor the regression test indicates any funnel plot asymmetry, with *p*-values of 0.8793 and 0.6559, respectively. Figure 5 illustrates the forest plot and other results for the meta-analysis assessing HDL-C levels throughout the included studies.

### 3.4. Results of the Meta-Analysis for Total Cholesterol

**Figure 6:**
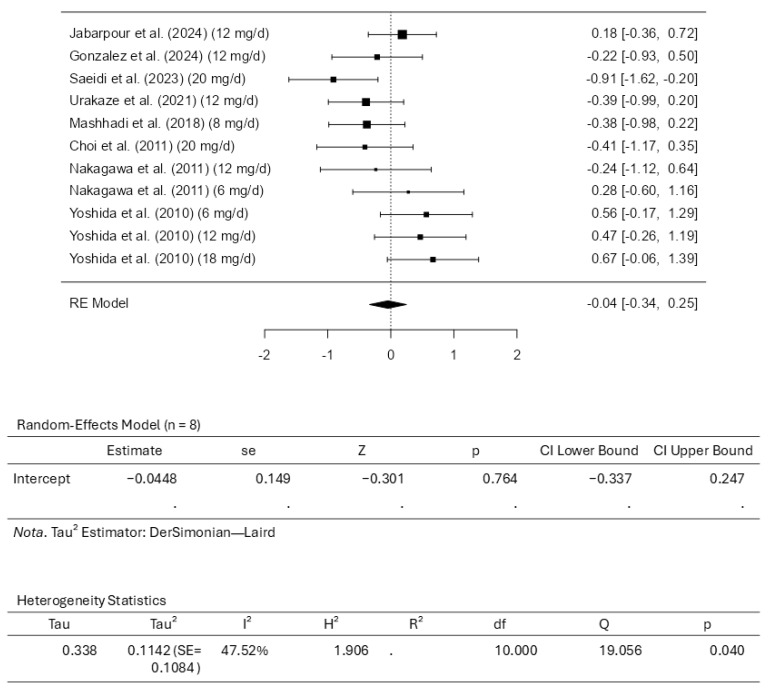
Forest plot depicting the sample setting for total cholesterol. The included studies are [27,28,29,30,31,32,33,34].

Eight studies comprising eleven results were included in the meta-analysis regarding total cholesterol intervention using astaxanthin. The standardized mean differences analysis ranged from −0.9100 to 0.6687; most estimates were negative (55%). While assessing the intervention, the estimated average standardized mean difference based on the random-effects model was calculated as −0.0448 (95% CI: −0.3369 to 0.2473). This means that the average outcome did not differ significantly from zero (z = −0.3007, *p* = 0.7636). Based on the results from the Q-test, it was observed that the outcomes appear to be heterogeneous, with statistical results from Q ranging from 19.0558, with a significance of *p* = 0.0396 (tau^2^ = 0.1142, I^2^ = 47.5225%). The 95% prediction interval for the outcomes was evaluated as a −0.7686 to 0.6790 interval. Therefore, although the average outcome is estimated to be negative, it may be positive in some studies. Examining the studentized residuals revealed that none of the studies included has a value larger than ±2.8376. This means there is no indication of outliers among the included studies. Cook’s distances reveal that no study included could be considered overly influential. Further analysis revealed that neither the rank correlation nor the regression test indicates asymmetry in the funnel plot (*p* = 0.3587 and *p* = 0.6918, respectively). Figure 6 illustrates the forest plot and other results for the meta-analysis assessing total cholesterol levels throughout the included studies.

### 3.5. Results of the Meta-Analysis for Triglycerides

**Figure 7:**
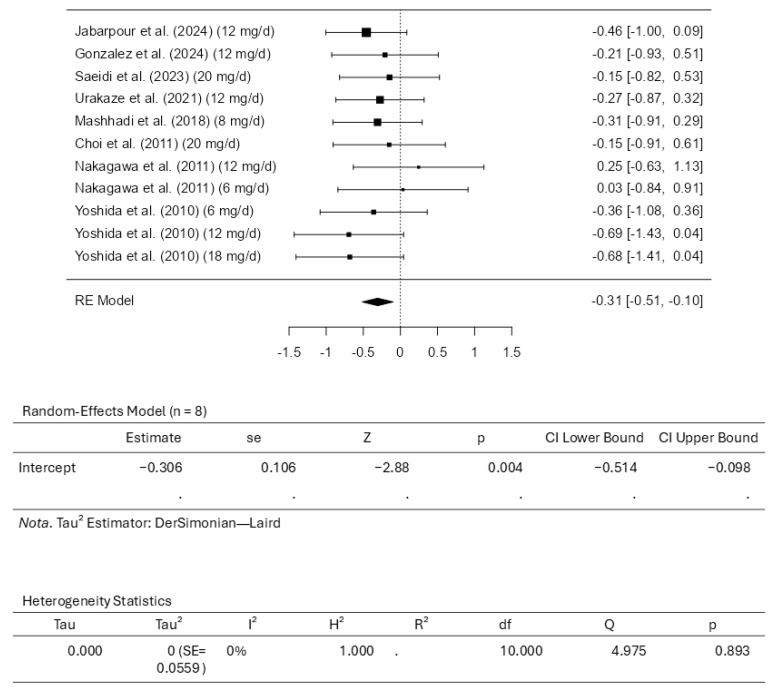
Forest plot depicting the sample setting for triglycerides. The included studies are [27,28,29,30,31,32,33,34].

Eight studies comprising eleven results were included in the meta-analysis assessing the triglyceride intervention with astaxanthin. A standardized mean difference ranged from −0.6942 to 0.2459, with most estimates being negative (82%). An estimated average standardized mean difference based on the random-effects model was calculated as −0.3058 (95% CI: −0.5138 to −0.0978). This means that the average outcome differed significantly from zero (z = −2.8816, *p* = 0.0040). According to the Q-test, the outcomes had no significant heterogeneity (4.9752, *p* = 0.8928, tau^2^ = 0.0000, I^2^ = 0.0000%). Examining the studentized residuals revealed that none of the studies has a value larger than ±2.8376, which means there is no indication of outliers. Cook’s distances reveal that none of the studies could be considered overly influential. Neither the rank correlation nor the regression test indicates any funnel plot asymmetry (*p* = 0.2830 and *p* = 0.3224, respectively). Figure 7 illustrates the forest plot and other results for the meta-analysis assessing triglyceride levels throughout the included studies.

### 3.6. Certainty Assessment

Table S1 summarizes the certainty of evidence from the eight randomized controlled trials evaluating the eleven outcomes and the effects of astaxanthin supplementation compared to placebo on lipid profile outcomes, including LDL-C, HDL-C, total cholesterol, and triglycerides.

LDL-C: Certainty: ⨁⨁◯◯—low

Reasons for downgrading: severe risk of bias due to inadequate randomization and lack of intention-to-treat analysis. The effect of astaxanthin on LDL-C is uncertain and likely minimal.

HDL-C: Certainty: ⨁◯◯◯—very low

Reasons for downgrading: severe risk of bias and high heterogeneity. Astaxanthin may increase HDL-C levels, but the certainty of the evidence is very low.

Total Cholesterol: Certainty: ⨁◯◯◯—very low

Reasons for downgrading: severe risk of bias and moderate heterogeneity. The effect of astaxanthin on total cholesterol is highly uncertain.

Triglycerides: Certainty: ⨁⨁◯◯—low

Reasons for downgrading: there is a severe risk of bias, although inconsistency is low and the estimates are precise. Astaxanthin may reduce triglyceride levels, though confidence in this result is limited.

### 3.7. Dose-Response Meta-Analysis for the Positive and Negative Outcomes on High-Density Lipoprotein Cholesterol and Triglyceride Levels and Total Cholesterol and Low-Density Lipoprotein Cholesterol, Respectively

**Figure 8:**
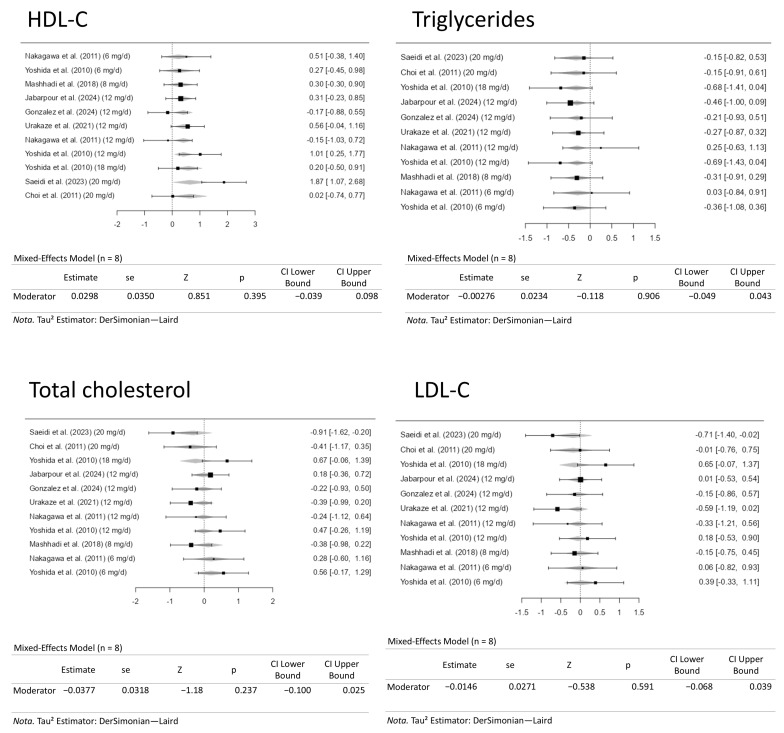
Dose-response meta-analysis for high-density lipoprotein cholesterol (HDL-C), triglyceride, low-density lipoprotein cholesterol (LDL-C), and total cholesterol levels. The included studies are [27,28,29,30,31,32,33,34].

A dose-response meta-analysis was conducted to evaluate the effects of moderate to high doses of astaxanthin (6 to 20 mg/d) on the positive (HDL-C and triglycerides) and negative (LDL-C and total cholesterol) outcomes in the present study. Figure 8 gives the forest plots from these analyses. Controlling the doses of the astaxanthin supplementation did not alter the outcome measure. Therefore, controlling the dose of the supplement did not alter the previously mentioned results.

## 4. Conclusions and Future Research Perspectives

**Figure 9:**
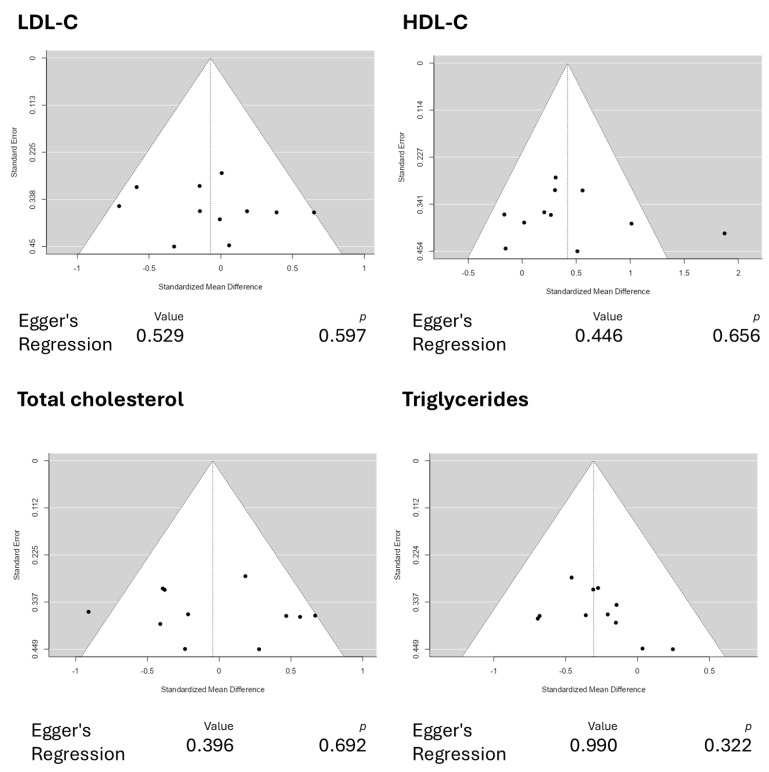
Funnel plot analyses for the reported outcomes. The funnel plots depict no plot asymmetry, indicating good reliability in the reported results. Abbreviations: HDL-C, high-density lipoprotein cholesterol; LDL-C, low-density lipoprotein cholesterol.

This updated systematic review and meta-analysis demonstrated positive and promising results regarding the modulation of HDL-C and triglyceride levels using astaxanthin as a functional supplement. The positive effects of astaxanthin in HDL-C modulation were marked by an average standardized mean difference of 0.4200 (95% CI: 0.1081 to 0.7319). In comparison, triglycerides were modulated with a −0.3058 (95% CI: −0.5138 to −0.0978) standardized mean difference, demonstrating significant results. Additionally, our funnel plot analyses reported no plot asymmetry, as depicted in Figure 9, indicating good reliability in the reported results.

Although the analysis was not promising for LDL-C and total cholesterol levels, some future research endeavors must be addressed to fully harness astaxanthin’s potential in modulating human lipid profiles, opening doors for future positive results in the overall lipid profile of patients. Firstly, researchers must elucidate the possible nuanced effects of astaxanthin in subpopulations. For example, the compound might possess differential effects while being assessed in patients suffering from metabolic syndrome, diabetes, or cardiovascular diseases such as stroke. Therefore, conducting research in various populations is vital to investigate the functional supplement’s effects and assess their effectiveness for different health statuses. In this scenario, conducting genome-wide association studies (GWAS) is also notable for its association with nutrigenomics. Nutrigenomics studies how food and dietary components influence gene expression with regard to genetic variants and other nutritional factors, and how specific food roles influence the genome at molecular levels, allowing for broad investigations into how food modulates the genetic expression of enzymes and other cellular functions to impact human health [35]. Therefore, studying how astaxanthin affects the human genome under varying conditions might be essential to assess its effects on lipid dysregulation in various situations, including obesity [36] or sarcopenia [37], which are also critical conditions related to cardiovascular health [38] in which lipid regulation would be greatly beneficial.

Furthermore, it would help to combine therapies using astaxanthin. For example, combining astaxanthin with other nutraceuticals, like omega-3s or plant sterols, which possess promising anti-dyslipidemic effects, may also result in synergistic effects on the complete lipid profile, thereby providing astaxanthin supplementation with a higher level of evidence. In this regard, icosapent ethyl, a well-studied omega-3 with potential as a lipid-lowering drug, could be an option for combination with astaxanthin due to its synergistic effects on inflammation, oxidative stress, and lipid peroxidation, which could not only diminish cholesterol levels but also decrease cardiovascular risk in patients [39]. Researchers must also combine other compounds with different formulations of astaxanthin to improve its solubility or bioavailability, including free, natural, or synthetic formulations.

Despite these research avenues, more well-designed, randomized, placebo-controlled clinical studies comprising thousands of participants of varying ages, genders, occupations, and nationalities must be conducted to assess astaxanthin more deeply regarding dose-responsiveness, tolerability, and pharmacoeconomics. By exploring these future research directions, the field of research will continue to advance, and, possibly, future generations of clinicians will prescribe astaxanthin as a lipid-lowering agent during routine clinical practice. Future clinical studies should also aim to elucidate the actual correlation between HDL-C improvement due to astaxanthin supplementation and a reduction in overall cardiovascular disease risk and outcomes. This would pave the way for astaxanthin to be recommended in guidelines of scientific organizations, including the ones on cardiovascular protection and treatment.

Although this study yields essential results, its meta-analysis has limitations that must be addressed. Firstly, it is necessary to note that, due to the potential for publication bias, the overall findings of this meta-analysis may be subject to an overestimation of effect sizes. However, this may happen with all meta-analyses. Additionally, some aspects of this meta-analysis presented substantial heterogeneity, including HDL-C (I^2^ = ~52.7%, indicating high heterogeneity) and total cholesterol (I^2^ = ~47.5%, indicating moderate heterogeneity), which may limit the confidence of the abovementioned results. Finally, the studies reported data on astaxanthin supplementation in subjects presenting various health conditions beyond dyslipidemia, which may be considered a limitation due to inconsistent grouping, potentially limiting the generalizability of the findings. However, the present meta-analysis also has many strengths. Firstly, this is the first review to assess the effectiveness of moderate to high dosages of astaxanthin supplementation in lipid levels using statistical tools. Secondly, due to our prominent search strategy and adherence to the PRISMA guidelines, this meta-analysis ensures that all relevant studies were included in the final analysis. Furthermore, since we identified possible future research directions based on our findings, we pinpointed areas where evidence is lacking and where a more sufficient background is needed, promoting the conduct of new, well-designed research in this area.

## Figures and Tables

**Table 1:** Report of the included studies following the PRISMA Guidelines [20].

Study	Local	Patients	Intervention	Outcomes	Adverse Effects	Observations
[29]	Iran	Triple-blind, randomized, placebo-controlled clinical trial with infertile women diagnosed with PCOS. Intervention group (age: 30.36 ± 5.16 years; BMI: 26.12 ± 1.56 kg/m^2^). Placebo group (age: 30.84 ± 4.84 years; BMI: 26.24 ± 1.59 kg/m^2^).	2 × 6 mg astaxanthin (27 patients) or a placebo (26 patients) was administered daily for 8 weeks.	TC: 172.8 ± 38.99 mg/dL → 169.1 ± 37.65 mg/dL (*p* = 0.106). TG: 180.9 ± 65.08 mg/dL → 180.4 ± 64.30 mg/dL (*p* = 0.224). HDL-C: 37.61 ± 10.08 mg/dL → 41.80 ± 9.965 mg/dL (*p* = 0.003). LDL-C: 91.27 ± 21.08 mg/dL → 82.52 ± 23.58 mg/dL (*p* = 0.013).	Patients experienced no side effects or symptoms associated with astaxanthin consumption.	The intervention group had 2 losses to follow-up, and the placebo group had 3.
[30]	USA	Double-blind, randomized, placebo-controlled, crossover fashion study with 15 healthy male career firefighters (age: 34.5 ± 7.5 years). Astaxanthin group: BMI 30.16 ± 3 kg/m^2^. Placebo group: 30.14 ± 2.9 kg/m^2^.	Participants received a placebo or 12 mg of astaxanthin daily for 4 weeks while participating in a standardized training program. They observed a 2-week washout and repeated the experiment with the alternative treatment for more 4 weeks.	TC: 197.8 ± 29.9 mg/dL → 217.9 ± 33.2 mg/dL (*p* = 0.783). TG: 117.7 ± 40.9 mg/dL → 132.5 ± 45 mg/dL (*p* = 0.827). HDL-C: 47.1 ± 8.4 mg/dL → 51.9 ± 9.3 mg/dL (*p* = 0.724). LDL-C: 127.7 ± 28.3 mg/dL → 140.5 ± 30.3 mg/dL (*p* = 0.628).	Some patients presented with dizziness, headache, tachycardia, palpitations, dyspnea, nervousness, and blurred vision. No significant differences were observed between treatments in perceptions of the frequency or severity of these effects.	20 subjects were randomized, but 5 dropped out after beginning the study.
[31]	Iran	Randomized, placebo-controlled, clinical trial involving men with obesity (age: 27.6 ± 8.4 years).	Participants were stratified into groups of 17 subjects each: a control group (BMI: 34.1 ± 2.5 kg/m^2^; body fat: 31.1 ± 1.5%; height: 167.5 ± 2.7 cm; weight: 95.3 ± 1.8 kg) and a supplement group (BMI: 33.2 ± 1.4 kg/m^2^; body fat: 31.1 ± 1.5%; height: 168.2 ± 3.5 cm; weight: 94.2 ± 2.6 kg). Participants underwent 12 weeks of treatment with astaxanthin 20 mg/day or placebo.	TC: 229.4 ± 5.4 mg/dL → 224.0 ± 5.1 mg/dL (*p* < 0.05). TG: 247.8 ± 5.9 mg/dL → 244.1 ± 5.4 mg/dL (*p* < 0.05). HDL-C: 37.8 ± 1.23 mg/dL → 39.8 ± 1.2 mg/dL (*p* < 0.05). LDL-C: 127.5 ± 5.4 mg/dL → 123.4 ± 5.2 mg/dL (*p* < 0.05).	Not reported.	During the experiment period, 8 participants from different groups withdrew from the study, leaving 15 participants in each group.
[32]	Japan	Double-blind, randomized, parallel, placebo-controlled trial involving healthy subjects. Intervention group (8 men and 15 women; age: 46.2 ± 13.7 years; BMI: 21 ± 2 kg/m^2^). Placebo group (7 men and 14 women; age: 48.2 ± 12 years; BMI: 23.9 ± 5.4 kg/m^2^).	12 mg astaxanthin (23 patients) or a placebo (21 patients) was administered once daily for 12 weeks.	TC: 199.4 ± 25.8 mg/dL → 200.3 ± 30.4 mg/dL (*p*-value was not reported). TG: 84.9 ± 39 mg/dL → 90.3 ± 53.7 mg/dL (*p*-value was not reported). HDL-C: 64 ± 14.3 mg/dL → 64.7 ± 15.3 mg/dL (*p*-value was not reported). LDL-C: 118.5 ± 22.1 mg/dL → 117.5 ± 24.2 mg/dL (*p*-value was not reported).	Patients experienced no side effects with astaxanthin consumption.	The intervention group had 4 losses to follow-up, and the placebo group had 5.
[33]	Iran	Double-blind, randomized, parallel, placebo-controlled trial involving subjects with T2DM. Intervention group (BMI: 30 ± 5.11 kg/m^2^; body fat: 35.5 ± 10.6%). Placebo group (BMI: 30.4 ± 5 kg/m^2^; body fat: 38.6 ± 9.8%).	8 mg astaxanthin (22 patients) or a placebo (21 patients) was administered once daily for 8 weeks.	TC: 153.8 ± 35 mg/dL → 146 ± 30 mg/dL (*p* = 0.06). TG: 156 ± 90 mg/dL → 128 ± 52 mg/dL (*p* = 0.05). HDL-C: 37.4 ± 5.2 mg/dL → 38.1 ± 5.7 mg/dL (*p* = 0.09). LDL-C: 85.7 ± 27 mg/dL → 88 ± 27 mg/dL (*p* = 0.54).	Patients experienced no side effects with astaxanthin consumption.	The study presented losses to follow-up.
[34]	South Korea	Double-blind, randomized, placebo-controlled study with 27 overweight subjects. Intervention group (12 men and 2 women; 31.1 ± 9.4 years; BMI: 28.1 ± 2.4 kg/m^2^; body weight: 83.6 ± 9.4 kg; height: 1.72 ± 0.07 m; waist circumference: 97.1 ± 6.3 cm). Placebo group (11 men and 2 women; age: 30.1 ± 9.5 years; BMI: 26.3 ± 1.3 kg/m^2^; body weight: 77.1 ± 10.8 kg; height: 1.71 ± 0.10 m; waist circumference: 92.1 ± 6.2 cm).	20 mg astaxanthin (14 patients) or a placebo (13 patients) was administered once daily for 12 weeks.	TC: 178.3 ± 3.54 mg/dL → 169.8 ± 3.19 mg/dL (*p*-value was not reported). TG: 110.6 ± 51.5 mg/dL → 110.9 ± 38.4 mg/dL (*p*-value was not reported). HDL-C: 47.2 ± 10.2 mg/dL → 50.4 ± 12.6 mg/dL (*p*-value was not reported). LDL-C: 127.9 ± 35 mg/dL → 114.6 ± 28.6 mg/dL (*p* < 0.05).	Fecal color changed to red, and an increase in bowel movements.	All participants completed the study.
[27]	Japan	Double-blind, randomized, placebo-controlled trial with 30 healthy subjects. Intervention group 6 mg (5 men and 5 women; age: 56.3 ± 6.6 years; BMI: 27.4 ± 2.2 kg/m^2^). Intervention group 12 mg (5 men and 5 women; age: 56.1 ± 5.1 years; BMI: 27.6 ± 2.1 kg/m^2^). Placebo group (5 men and 5 women; age: 56.6 ± 4.4 years; BMI: 27.7 ± 2.1 kg/m^2^).	6 mg astaxanthin (10 patients), 12 mg astaxanthin (10 patients), or a placebo (10 patients) was administered once daily for 12 weeks.	6 mg group → TC: 226 ± 39 mg/dL → 215 ± 32 mg/dL (*p*-value was not reported). TG: 111 ± 78 mg/dL → 116 ± 67 mg/dL (*p*-value was not reported). HDL-C: 68.8 ± 19 mg/dL → 65.5 ± 14.7 mg/dL (*p*-value was not reported). LDL-C: 132 ± 37 mg/dL → 124 ± 29 mg/dL (*p*-value was not reported). 12 mg group → TC: 203 ± 23 mg/dL → 196 ± 15 mg/dL (*p*-value was not reported). TG: 125 ± 73 mg/dL → 136 ± 114 mg/dL (*p*-value was not reported). HDL-C: 58.8 ± 12.1 mg/dL → 56.2 ± 10.5 mg/dL (*p*-value was not reported). LDL-C: 117 ± 20 mg/dL → 112 ± 19 mg/dL (*p*-value was not reported).	Not reported.	All participants completed the study.
[28]	Japan	Double-blind, randomized, placebo-controlled study with 61 moderately hypertriglyceridemic subjects. Intervention group 6 mg (10 men and 5 women; age: 47 ± 7 years; BMI: 23.6 ± 3.2 kg/m^2^). Intervention group 12 mg (10 men and 5 women; age: 42.8 ± 8.8 years; BMI: 23 ± 2.2 kg/m^2^). Intervention group 18 mg (11 men and 5 women; age: 43.8 ± 10.4 years; BMI: 23.9 ± 7 kg/m^2^). Placebo group (10 men and 5 women; age: 44.3 ± 7 years; BMI: 25.1 ± 2.8 kg/m^2^).	The participants were allocated to four groups with a 12-week treatment of 6 mg/day (15 patients), 12 mg/day (15 patients), and 18 mg/day (16 patients) of astaxanthin or placebo (15 patients).	6 mg group → TC: 219 ± 29 mg/dL → 228 ± 30 mg/dL (*p*-value was not reported). TG: 151 ± 23 mg/dL → 125 ± 41 mg/dL (*p* < 0.05). HDL-C: 51 ± 11 mg/dL → 56 ± 11 mg/dL (*p* < 0.01). LDL-C: 141 ± 26 mg/dL → 150 ± 28 mg/dL (*p*-value was not reported). 12 mg group → TC: 215 ± 22 mg/dL → 225 ± 29 mg/dL (*p*-value was not reported). TG: 147 ± 21 mg/dL → 110 ± 44 mg/dL (*p* < 0.05). HDL-C: 55 ± 8 mg/dL → 63 ± 8 mg/dL (*p* < 0.01). LDL-C: 136 ± 27 mg/dL → 144 ± 32 mg/dL (*p*-value was not reported). 18 mg group → TC: 234 ± 29 mg/dL → 233 ± 35 mg/dL (*p*-value was not reported). TG: 151 ± 26 mg/dL → 112 ± 40 mg/dL (*p* < 0.01). HDL-C: 51 ± 6 mg/dL → 55 ± 8 mg/dL (*p* < 0.05). LDL-C: 157 ± 25 mg/dL → 159 ± 31 mg/dL (*p*-value was not reported).	Not reported.	All participants completed the study.

Abbreviations: BMI, Body Mass Index; HDL-C, high-density lipoprotein cholesterol; LDL-C, low-density lipoprotein cholesterol; PCOS, polycystic ovary syndrome; T2DM, type 2 diabetes mellitus; TC, total cholesterol; TG, triglycerides.

**Table 2:** Report of bias identification throughout the studies following the *Cochrane Handbook for Intervention Assessment* [21].

Study	Question Focus	Appropriate Randomization	Allocation Blinding	Double-Blind	Losses (<20%)	Prognostic or Demographic Characteristics	Outcomes	Intention-to-Treat Analysis	Sample Calculation	Adequate Follow-Up
[29]	Yes	No	Yes	Yes	Yes	Yes	Yes	No	Yes	Yes
[30]	Yes	NR	Yes	Yes	No	Yes	Yes	Yes	NR	Yes
[31]	Yes	No	No	No	Yes	Yes	Yes	Yes	Yes	Yes
[32]	Yes	No	Yes	Yes	No	Yes	Yes	No	NR	Yes
[33]	Yes	NR	Yes	Yes	Yes	Yes	Yes	Yes	NR	Yes
[34]	Yes	NR	Yes	Yes	Yes	Yes	Yes	Yes	Yes	Yes
[27]	Yes	NR	Yes	Yes	Yes	Yes	Yes	Yes	NR	Yes
[28]	Yes	NR	Yes	Yes	Yes	Yes	Yes	Yes	NR	Yes

Abbreviation: NR, not reported.

## Data Availability

The raw data supporting the conditions of this meta-analysis will be made available for readers upon reasonable request to the corresponding author.

## Supplementary Materials

The following supporting information can be downloaded at https://www.mdpi.com/article/10.3390/ph18081097/s1. The raw data supporting the conditions of this meta-analysis will be made available for readers upon reasonable request to the corresponding author. Figures S1 and S2 and Table S1 can be found in the Supplementary Material associated with this article.
