# Supplementary material for: Assessing the Effects of Moderate to High Dosage of Astaxanthin Supplementation on Lipid Profile Parameters—A Systematic Review and Meta-Analysis of Randomized Controlled Studies
Source: Pharmaceuticals (Basel). 2025 Jul 24;18(8):1097. doi: 10.3390/ph18081097 (PMC12389351; doi:10.3390/ph18081097)
Supplement: Supplementary file 1 [file pharmaceuticals-18-01097-s001.zip › Table S1.pdf]

Author(s):
Question: Astaxanthin compared to Placebo for lipid profile
Setting:
Bibliography:

Certainty assessment							№ of patients		Effect		Certainty	Importance
№ of studies	Study design	Risk of bias	Inconsistency	Indirectness	Imprecision	Other considerations	Astaxanthin	Placebo	Relative (95% CI)	Absolute (95% CI)		
**LDL**												
11	randomised trials	very serious[a,b]	not serious	not serious	not serious	none			-	SMD **0.07 SD lower** (0.31 lower to 0.16 higher)	⨁⨁◯◯ Low[a,b]	
**HDL**												
11	randomised trials	very serious[a,b]	serious[c]	not serious	not serious	none			-	SMD **0.42 SD higher** (0.11 higher to 0.73 higher)	⨁◯◯◯ Very low[a,b,c]	
**Total cholesterol**												
11	randomised trials	very serious[a,b]	serious[d]	not serious	not serious	none			-	SMD **0.04 SD lower** (0.34 lower to 0.25 higher)	⨁◯◯◯ Very low[a,b,d]	
**Triglycerides**												
11	randomised trials	very serious[a,b]	not serious	not serious	not serious	none			-	SMD **0.31 SD lower** (0.51 lower to 0.1 lower)	⨁⨁◯◯ Low[a,b]	

CI: confidence interval; **SMD:** standardised mean difference

Explanations

a. Not Appropriate Randomiz ation
b. Intention to Treat Analysis
c. I2=52.7%.
d. I2-47.5%
