# Supplementary figures and images for: Assessing the Effects of Moderate to High Dosage of Astaxanthin Supplementation on Lipid Profile Parameters—A Systematic Review and Meta-Analysis of Randomized Controlled Studies
Source: Pharmaceuticals (Basel). 2025 Jul 24;18(8):1097. doi: 10.3390/ph18081097 (PMC12389351; doi:10.3390/ph18081097)

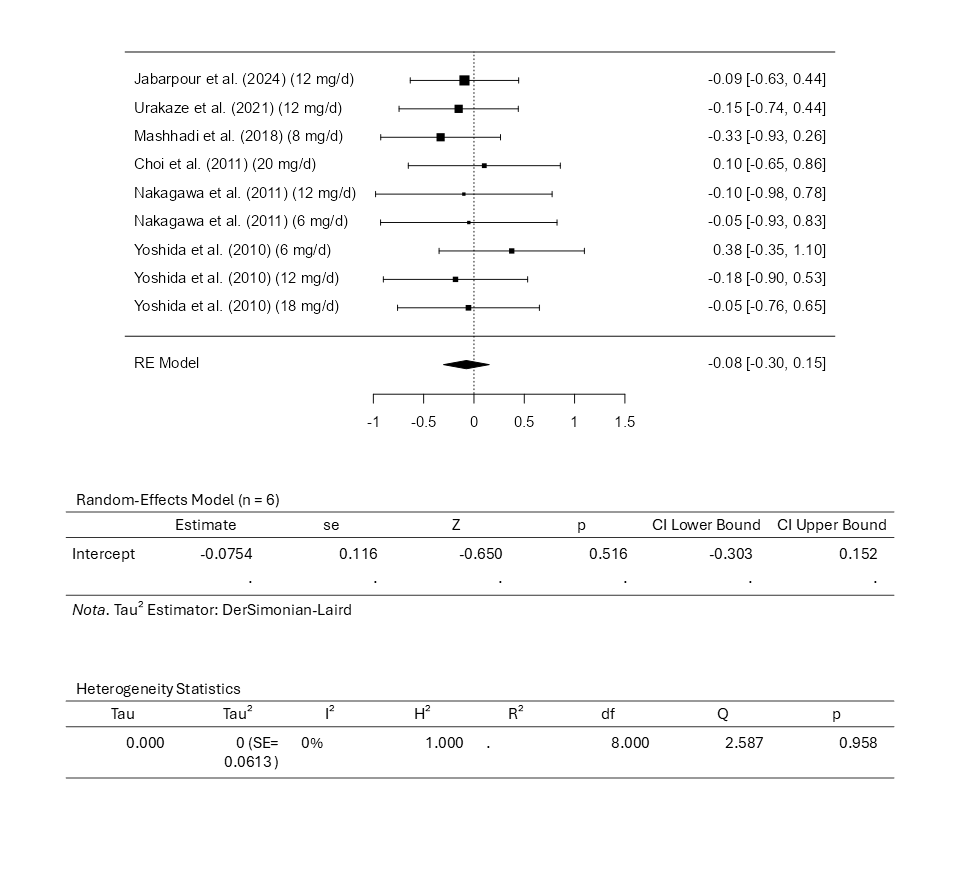

Supplement: Supplementary file 1 [file pharmaceuticals-18-01097-s001.zip › Figure S1_Age OK.tif]

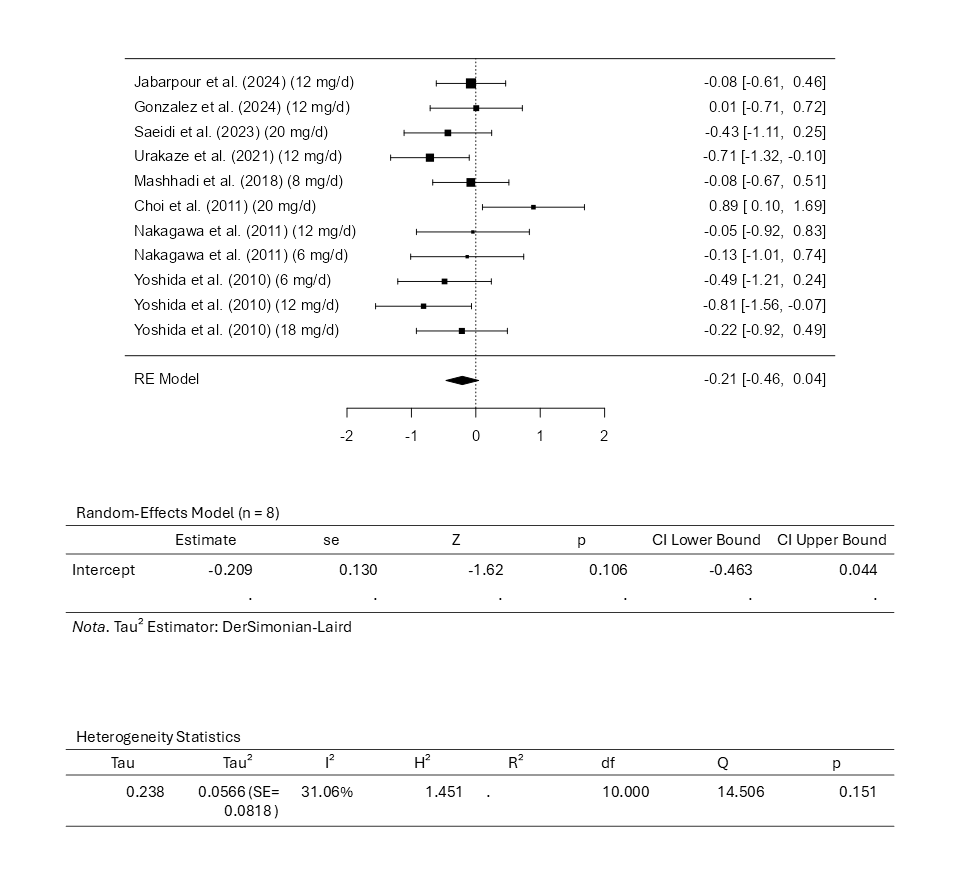

Supplement: Supplementary file 1 [file pharmaceuticals-18-01097-s001.zip › Figure S2_BMI OK.tif]
